# Parliament and the Professions

**Published:** 1920-08-07

**Authors:** 


### Parliament and the Professions.

### National Health Referee Consultants.

Dr. Addison informed Sir J. D. Rees that the thirty-two medical men recently appointed under the National Health Insurance scheme as referee consultants have the duty of protecting insurance funds from unnecessary claims to sickness benefit and of improving the general standard of insurance medical practice. Funds for the purpose were voted in 1914, but were not spent during the war owing to impossibility of establishing this much-needed extension of the Health Insurance Medical Service during that time.

### Health of Mesopotamia Troops.

Mr. Churchill stated that for the first three months of this year the health of the British troops in Mesopotamia was very satisfactory, the sick rate being less than that in the United Kingdom. In April, the last month for which complete figures are available, the admission rate per 1,000 of strength rose owing to the seasonal incidence of sandfly fever, giving an annual ratio of 890.4 per 1,000, compared with 664.6 per 1,000 in the United Kingdom. The incidence of sandfly fever increased during May and June, but is now declining rapidly.

### Nursing Staffs of Prison Hospitals.

The Home Secretary stated that before an officer can be appointed to the hospital staff of a prison he or she must undergo a special course of training at a prison hospital training-school and must be reported as fit in all respects for the duties. These hospital staff officers are all permanent officials and come under the Superannuation Acts. A staff is in course of formation at Holloway Prison, and will consist of a lady superintendent, two principal nurses, and forty-eight nurses.

